# Propolis as an alternative remedy for the treatment of subclinical mastitis in dairy cows

**DOI:** 10.3389/fvets.2025.1740383

**Published:** 2026-02-02

**Authors:** Zoja Mikniene, Grazvydas Puska, Mindaugas Liaudanskas, Jurate Siugzdaite, Loreta Kubiliene, Jurate Rudejeviene, Vaidotas Zvikas, Neringa Sutkeviciene, Ona Ragazinskiene, Sonata Trumbeckaite

**Affiliations:** 1 Veterinary Academy, Lithuanian University of Health Sciences, Kaunas, Lithuania; 2 Medicine Academy, Lithuanian University of Health Sciences, Kaunas, Lithuania; 3 Kaunas Botanical Garden of Vytautas Magnus University, Kaunas, Lithuania

**Keywords:** antioxidant activity, cattle, flavonoids, phenolic acids, propolis, subclinical mastitis

## Abstract

**Background:**

Subclinical mastitis is a widespread condition in dairy cows, often treated with antibiotics. Due to rising antimicrobial resistance, natural alternatives like propolis are gaining interest.

**Objectives:**

To evaluate the antibacterial efficacy of 5 and 10% propolis alginate emulsions in cows with subclinical mastitis and assess their effects on milk quality and systemic biomarkers.

**Methods:**

Ten dairy cows diagnosed with subclinical mastitis were divided into two treatment groups (5 and 10% propolis emulsions). Emulsions were administered intramammarily and orally for 5 days. Bacterial load in milk, milk composition, blood biochemical and immunological parameters, phenolic compound profiles, and antioxidant activity were analysed using standard microbiological and biochemical methods.

**Results:**

The 5% emulsion reduced bacterial load by 2.27 log CFU/ml, outperforming the 10% emulsion (0.89 log CFU/ml; *p* < 0.05). Significant changes were observed in hepatic enzymes (ALT, AST), electrolytes (Ca, Mg, P), and renal markers (creatinine, urea). Immunological shifts included an increase in lymphocytes (+21%) and a decrease in neutrophils (−30%) in the 5% group. Antioxidant capacity was confirmed, with the highest activity observed in CUPRAC and ABTS assays. Phenolic compounds, including p-coumaric acid and flavonoids, contributed to the bioactivity.

**Conclusion:**

A 5% propolis alginate emulsion was more effective than the 10% formulation in reducing bacterial counts and improving immunological and biochemical profiles. These findings support propolis as a promising natural alternative for treating subclinical mastitis, warranting further investigation into optimal formulations and long-term safety.

## 1 Introduction

Mastitis, an inflammation of the mammary gland, is a prevalent issue in dairy cows, especially in high-yield herds. Its complex aetiology involves interactions between the host, microorganisms, environment, and farming conditions (1). Subclinical mastitis is more frequent than clinical mastitis—by 3 to 40 times—and is a major source of economic loss (2). Though it does not alter milk appearance, it is marked by increased somatic cell count, chemical changes, and the presence of pathogens (3, 4).

Antibiotics remain the primary treatment, but their effectiveness is inconsistent, and overuse contributes to antimicrobial resistance. WHO recognizes this resistance as a global health concern and promotes strategies to mitigate it (5). Many veterinarians consider subclinical mastitis an area for improved antibiotic use (1, 3, 6).

Propolis, a natural bee product, has been used for centuries and is known as a “natural antibiotic” (7, 8, 46, 47, 49). It contains various bioactive compounds with antibacterial, antifungal, antiviral, anti-inflammatory, and antioxidant properties (1, 9–11, 68, 71) and is considered a potential alternative to antibiotics (12, 13, 69). The antimicrobial effects of propolis against common mastitis pathogens, the anti-inflammatory and antioxidant effects, which may support udder tissue recovery and modulate local immune responses (14–16, 50).

However, propolis has poor water solubility—only up to 5% in water versus 50–70% in ethanol. This is due to its hydrophobic components, like flavonoids and terpenoids. Solubilizing agents such as macrogol enhance dispersion and bioavailability, making propolis more usable in formulations like sprays and ointments.

This study aims to evaluate the antibacterial efficacy of various propolis-alginate solution concentrations in milk and to investigate the phytochemical composition and antioxidant activity of the most effective formulation *in vitro*. Our study addresses the lack of *in vivo* data on alginate-based, water-dispersible propolis formulations to evaluate the antibacterial effect of 5 and 10% propolis for intramammary treatment of subclinical mastitis in dairy cows and to characterize the phenolic profile and antioxidant activity of the most promising formulation. This topic is timely in the current AMR, animal welfare, and antibiotic reduction context.

## 2 Materials and methods

### 2.1 Propolis raw material and preparation of propolis alginate solution

Type of propolis poplar-type/brown propolis, based on local flora and our previous chemical characterization. Raw propolis (*Apis mellifera carnica*) was collected in Lithuania during late summer to early autumn (August–September) from regions dominated by Populus spp. and other temperate flora. Samples free of visible impurities were manually cleaned prior to storage and further processing. Propolis emulsions of different concentrations (5 and 10%) and oral aqueous propolis extract (10%) were prepared at the Medical Academy of Lithuanian University of Health Sciences, Faculty of Pharmacy, Department of Pharmacognosy. The extraction was carried out according to a methodology adapted from (17). Crude propolis was ground into powder. Propolis emulsions of different concentrations were prepared by preparing 5 and 10% raw propolis with a mixture of 20% polyethylene glycol 400 (makrogolum) and water [the sample to solvent ratio 1: 10 (water + PEG 400)] using ultrasonic extraction, and after adding sodium alginate (2%). Propolis powder was dispersed in a polyethylene glycol 400–distilled water mixture, ultrasonicated (35 kHz, 70 °C, 10 min), filtered through a defined pore-size filter, mixed with sodium alginate (2%, w/v) under stirring, and homogenized. Sodium alginate was chosen for its gel-forming and mucoadhesive properties, intended to prolong the contact time of active compounds with the teat canal and gland cistern. The 5 and 10% concentrations were selected based on solubility limitations; preliminary *in vitro* screening indicated that this range achieved adequate biological activity while maintaining acceptable viscosity and injectability. Oral 10% propolis extract was prepared by 10% raw propolis by adding the system of solvents—a mixture of water and 20% polyethylene glycol (PEG) 400 (makrogolum). The sample to solvent ratio 1: 10 (water + PEG 400). Extractions were carried out for 10 min at 70 °C temperature in the ultrasonic bath using 35 kHz frequency.

### 2.2 Animals and samples

The research was carried out in accordance with the provisions of the Law on Animal Welfare and Protection of the Republic of Lithuania [22 September Directive 2010/63/EU of the European Parliament and of the Council on the protection of animals used for scientific purposes (OJ 2010 L 276, p. 33)].

The study was carried out on a dairy farm in the Kaunas region of Lithuania (54.9753923, 23.7662303), involving a herd of 512 Lithuanian Black and White dairy cows, all 4 years old and in their first or second lactation. The California mastitis test (CMT), which detects both clinical and subclinical mastitis symptoms, was used to test all cows. The study included 10 clinically healthy Lithuanian Black and White dairy cows, aged one to two lactations, untreated for mastitis or other diseases, and showing elevated milk electrical conductivity along with a positive CMT result according to herd management protocol (55). Cows were selected based on positive CMT, elevated electrical conductivity, and SCC > 200,000 cells/mL in at least one quarter. These cows were divided equally into two groups before treatment (one group for 5% and the second for 10% of propolis treatment). The average body weight of all cows was 649.14 ± 18.92 kg. Cows were housed in loose housing conditions and fed a total mixed ration (TMR) twice daily. The diet was formulated to meet the nutritional requirements of high-yielding lactating Holstein cows and was supplemented with concentrates provided via an automatic milking system.

### 2.3 Milk sample collection and analysis

Samples were obtained in accordance with the guidelines of the US National Mastitis Council. The individuals’ udders were thoroughly cleansed with plenty of clean water and dried with paper towels, while the teats were disinfected using swabs saturated with 80% ethanol. Following the elimination of the initial milk stream, roughly 40 mL of quarter milk was gathered into 50 mL sterilised tubes and preserved at 4 °C till the hygiene analysis was conducted at the reference laboratory of the Milk Quality Control Foundation, Pieno tyrimai, within the hour after collection.

### 2.4 Somatic cell count

To perform the CMT test, 2–3 mL of milk from each quarter of the cow’s udder were used for elevated SCC (the somatic cell count) (*n* = 40). The somatic cell count was assessed using the fluoro-opto-electronic method on a Fossomatic 90 Mastitis & Milk Quality Tester/Somatic Cell Counter (Foss, Denmark), in accordance with the HRN ISO 133662:2006 standard. A cell count of 100,000 cells/mL was deemed a modest somatic cell count, as it is often indicative of a healthy mammary gland. A high somatic cell count (SCC) is defined as over 200,000 cells/mL, which serves as a logical threshold for indicating subclinical mastitis infection.

### 2.5 Microbiological analysis of milk

From each tested animal’s teats in sterile flasks were taken two 40–45 mL milk samples after cleaning of teat with 70% alcohol and disposing of the first three jets to microbial isolation. The pathogen investigation (genus or species identification) for the positive animals was carried out in the Microbiology Laboratory of the Lithuanian University of Health Sciences by culturing on media (Muller–Hinton, blood, and MacConkey agar). Incubation of the plates was conducted at 37 °C for 24 h in aerobic conditions. Morphological study of colonies and bacterial cells was conducted using Gramme stain following incubation. Preliminary tests were conducted for each colony to determine the appropriate analytical profile index (API) micro gallery type.

For bacteria count tenfold dilution was performed: 1 mL of milk and 9 mL of physiological solution. 1 mL of diluted sample was placed in Petri plate with MILK plate count agar (Oxoid, England) and kept in 48 h + 30 °C temperature under aerobic conditions. After the period bacteria in plates were counted in the range of 15–300 colony-forming units (CFU), under or over mentioned numbers of plates were not counted. The total count of microorganisms was determined according to the LST EN ISO 4833:2003 “Microbiology of food and feed.” General method. Colony count at 30 °C standard.

The quality of milk was evaluated by the State Enterprise “Pieno tyrimai.” The State Enterprise “Pieno tyrimai” is accredited to conduct physical and chemical analyses on raw milk. Milk samples were obtained in specialised 50 mL containers provided by “Pieno tyrimai.” Milk samples were delivered to the laboratories within 2 h at a temperature of not more than +10 °C.

### 2.6 Experimental testing

Udder condition was assessed using the Boumatic Robotics herd management system, which monitors milk electrical conductivity during milking. Values exceeding 12 mS/cm triggered a potential mastitis alert. Suspected cows were further tested using the California Mastitis Test (CMT), and all selected cows were CMT-positive. No animals showed systemic signs such as fever, udder swelling, or appetite loss.

Ten cows diagnosed with subclinical mastitis were divided into two groups. One group received 5 mL of 5% propolis emulsion per affected teat, while the other received 5 mL of 10% emulsion. Volumes per teat (5 mL) were chosen to match the physical capacity of the teat cistern and common intramammary dosing practices. Additionally, both groups were administered 40 mL of 10% propolis emulsion orally, diluted in distilled water. Treatment was conducted twice daily for five consecutive days, immediately after milking by Boumatic Robotics equipment.

Before treatment, all cows were tested for allergic sensitivity to propolis emulsions by dermal application. No allergic reactions were observed with either 5% or 10% formulations.

### 2.7 Milk and blood samples collection and testing

Milk and blood samples were collected from each cow before treatment, during treatment 42 h, and after treatment 120 h per quarter cow. Blood was drawn from the coccygeal vein using EDTA tubes for leukogram analysis and plain tubes for serum preparation. Samples were transported to the laboratory within 2 h, and serum was separated by centrifugation at 3000 rpm for 5 min. One milliliter of serum per sample was frozen at −20 °C until analysis.

Biochemical parameters, including urea, AST, ALT, ALP, iron, creatinine, triglycerides, calcium, potassium, magnesium, phosphorus, and total protein, were analysed using a SELECTRA Junior biochemistry analyser (Netherlands, 2006) with Spinreact reagents (Spain). Blood smears were stained with Haematocolor and analysed under 100 × magnification using immersion oil. Leukocyte differentiation was performed by counting 100 cells per smear.

Additionally, milk yield and electrical conductivity were monitored using Boumatic Robotics milking equipment over 5 days before, during, and after treatment.

### 2.8 UHPLC-ESI-MS/MS conditions

The qualitative and quantitative analysis of phenolic compounds was performed according to the previously validated and described UHPLC-ESI-MS/MS method (18).

Before the UPLC-ESI-MS/MS analysis, the extract was filtered through membrane filters (pore size 0.22 μm) produced by Carl Roth GmbH (Carl Roth GmbH & Co. KG, Karlsruhe, Germany).

### 2.9 Evaluation of antioxidant activity

#### 2.9.1 DPPH assay

The DPPH solution in 96.3% v/v ethanol (3 mL, 6 × 10^−5^ M) was mixed with 10 μL of the extracts. A decrease in absorbance was determined at *λ* = 517 nm using a UV/VIS spectrophotometer (Model i3, Hanon, Shandong, China) (19). This spectrophotometer was employed in the case of all antioxidant-related experiments. The results of all antioxidant tests are expressed as μmol Trolox equivalent (TE) per gram.

#### 2.9.2 ABTS assay

Three milliliter of ABTS^•+^ solution was mixed with 10 μL of the extracts. A decrease in absorbance was measured at *λ* = 734 nm (20).

#### 2.9.3 TFPH assay

Three milliliter of TFPH^•+^ solution was mixed with 10 μL of the extracts. A decrease in absorbance was measured at *λ* = 502 nm (21).

#### 2.9.4 CUPRAC assay

The CUPRAC solution encompassed CuCl_2_ × 2H_2_O (0.01 M in water), ammonium acetate buffer solution (0.001 M, pH = 7), and neocuproine (0.0075 M in ethanol) (1:1:1 ratio). Three milliliter of CUPRAC reagent was mixed with 10 μL of the extracts. An increase in absorbance was recorded at *λ* = 450 nm (22).

#### 2.9.5 FRAP assay

The FRAP solution encompassed TPTZ (0.01 M dissolved in 0.04 M HCl), FeCl_3_ × 6H_2_O (0.02 M in water), and acetate buffer (0.3 M, pH 3.6) (1:1:10 ratio). Three milliliter of freshly prepared FRAP reagent was mixed with 10 μL of the extracts. An increase in absorbance was recorded at *λ* = 593 nm (23). The natural extracts are multicomponent and may act through different antioxidant mechanisms. These *in vitro* assays are used to characterize the formulation, not to directly predict clinical outcomes.

### 2.10 Statistical analysis

Statistical analyses were performed using SPSS Statistics version 25 (IBM Corp., USA). Data are presented as mean ± standard deviation (SD). Due to the limited sample size, statistical analyses were primarily exploratory; differences between groups were assessed using one-way analysis of variance (ANOVA), followed by the least significant difference (LSD) *post hoc* test. Statistical significance was set at *p* < 0.05.

## 3 Results

### 3.1 Effect of propolis alginate solution on pathogens and blood parameters

In all cases, not only Gram-positive bacteria were detected (Table 1). In 7 out of 10 cows, one bacterial species was identified, in four cows, co-infection with two species was discovered, and in one cow, co-infection with three species was found.

**Table 1:** Pathogens identified in subclinical mastitis milk.

Identified microorganism	Gram +_/_−_	Positive milk samples	
		N	Percentage^a^, %
*Staphylococcus auricularis*	+	4	40
*Staphylococcus pseudintermedius*	+	2	20
*Escherichia coli*	−	2	20
*Staphylococcus aureus*	+	2	20
*Bacillus*	+	1	10

a The proportion of milk samples in which bacteria were identified.

The first group of cows, treated with 5% propolis emulsion, exhibited a substantial reduction in bacterial colony-forming units (CFU), reaching an effectiveness of 99.46% (*p* > 0.05). The bacteria isolated from the milk samples of this group of cows were *Staphylococcus auricularis* (treatment efficiency was 99.85%), *Staphylococcus aureus* (treatment efficiency was 99.96%), *Bacillus* spp. (Treatment efficiency was 97.81%) While in the second group of cows, the average effectiveness of treatment was 86.99% (*p* > 0.05). The bacteria isolated from the milk samples of the second group of cows were *Staphylococcus pseudintermedius* (treatment efficiency was 74.99%), *Escherichia coli* (treatment efficiency was 100.00%), and *Staphylococcus auricularis* (treatment efficiency was 85.00%).

The results of the study revealed that 5% propolis emulsion reduced the number of bacteria isolated from cows’ milk by 2.27 log CFU/ml (*p* < 0.05). Meanwhile, the efficiency of 10% propolis emulsion in reducing the number of bacteria was 0.89 log CFU/ml (*p* < 0.05).

Comparing milk yield, the group of cows treated with 5% propolis experienced an increase of 1.83% following treatment, whereas the 10% propolis group showed a 12% decrease (*p* < 0.05). The active acidity of milk in the 5% propolis group decreased on average by 0.88% after treatment (*p* > 0.05). In the 10% propolis group, the decrease in active acidity was 0.78% (*p* > 0.05). An increase in milk fat (0.93%) and lactose (1.32%) content was observed in this group following treatment.

Treatment also led to notable changes in several biochemical and morphological parameters. ALT and AST showed a strong mutual correlation, reinforcing their roles as key indicators of hepatic function. Electrolyte parameters such as calcium, magnesium, and phosphorus were closely interrelated, reflecting systemic mineral homeostasis. Creatinine and urea, both indicators of renal function, exhibited a strong positive correlation. Interestingly, GGT was negatively correlated with HDL, suggesting a possible link between hepatic activity and lipid metabolism.

Immunological changes were also evident: lymphocyte counts increased significantly in both groups (5% group: +21%, 10% group: +14%), while neutrophil levels declined markedly (−30% and −27%, respectively). Additionally, eosinophil levels doubled in the 10% group, potentially indicating a hypersensitivity response to the higher propolis concentration.

### 3.2 Identification and quantification of phenolic compounds of propolis alginate solution by UHPLC-ESI-MS/MS

Thirteen different phenolic compounds were identified and quantified by UHPLC-ESI-MS/MS in the propolis samples, with the qualitative and quantitative composition presented in Table 2 and the mass spectrometry parameters listed in Table 3. The total phenolic compound content of the 5% propolis extract was found to be 157.30 mg/.

**Table 2:** Phenolic compounds (mg/g) identified in 5% propolis alginate solution.

Compounds	Amount (mg/g)
3,4-Dihydroxybenzoic acid	2.05
p-Coumaric acid	107.60
Total phenolic acids	109.65
Hyperoside	1.84
Quercitrin	2.20
Isorhamnetin	2.36
Kaempherol	1.36
Pinobanksin	0.80
Total flavonols (including flavanonols)	8.56
Acacetin	0.92
Apigenin	3.08
Biochanin A	0.45
Total flavones (including isoflavones)	4.45
(−)-Epicatecin	30.28
(+)-Catechin	2.92
Total flavan-3-ols	33.20
Pinocembrin	1.44
Pinobanksin	0.80
Total phenolics	157.30

**Table 3:** Mass spectrometry parameters for the analysis of phenolic compounds.

Compound	Parent ion (*m*/*z*)	Daughter ion (*m*/*z*)	Cone voltage (V)	Collision chamber energy (*e*V)
3,4-Dihydroxybenzoic acid	153	81	40	25
p-Coumaric acid	163	93	28	22
Pinocembrin	255	211	100	20
Apigenin	269	117	54	36
Pinobanksin	271	197	30	20
Acacetin	283	63	30	40
Biochanin A	283	210	30	40
Kaempherol	285	185	50	25
(−)-Epicatecin	289	123	60	34
(+)-Catechin	289	123	60	34
Isorhamnetin	315	300	44	22
Quercitrin	447	300	50	26
Hyperoside	463	300	50	26

Two phenolic acids were detected in the propolis samples: 3,4-Dihydroxybenzoic acid, belonging to the hydroxybenzoic acid group, and p-coumaric acid, belonging to the hydroxycinnamic acid group. The flavanol group is the most abundant of the phenolic compounds identified and quantified in the propolis samples, with 5 identified compounds: the aglycones kaempferol and isorhamnetin, the quercetin glycosides hyperoside and isoquercitrin, and the aglycone pinobanksin, which is part of the subgroup of flavanonols.

The flavanone pinocembrin and 3 isoflavone derivatives were detected in the propolis samples analysed: apigenin, its methylated derivative acacetin (4′-Methoxyapigenin), and the isoflavone subgroup of genistein, the methylated derivative of genistein biochanin A (4′-Methylgenistein).

### 3.3 Determination of antioxidant activity in propolis alginate solution

The antioxidant activity of 5% propolis alginate solution was assessed using five spectrophotometric methodologies: ABTS, DPPH, TFPH (free radical scavenging assays), and CUPRAC, FRAP (reducing power assays). The results are summarized in Table 4.

**Table 4:** Antioxidant activity of 5% propolis alginate solution.

Assay	Antioxidant activity *in vitro* (mmol TE/g)
ABTS	4.98 ± 0.02
DPPH	0.97 ± 0.08
TFPH	0.59 ± 0.04
CUPRAC	9.37 ± 0.24
FRAP	3.67 ± 0.33

The results of this study indicate that a 5% propolis emulsion is more effective than a 10% concentration in reducing subclinical mastitis indicators and modulating immune response in dairy cows. These findings support propolis as a promising natural alternative for mastitis treatment, though further research is needed to optimize formulation and assess long-term safety.

## 4 Discussion

This study demonstrated a reduction in bacterial load in milk following treatment with propolis-based formulations. The identified pathogens are consistent with those commonly reported in subclinical mastitis, and the observed antibacterial effects are in line with previous studies describing the activity of propolis against both Gram-positive and Gram-negative bacteria (12, 24). However, given the exploratory nature of the study, the absence of control groups, and the limited sample size, these findings should be interpreted as preliminary rather than as definitive evidence of therapeutic efficacy. Differences observed between the two treatment groups suggest that formulation-related factors may influence treatment outcomes and warrant further investigation.

Milk yield and composition showed moderate changes following treatment. Previous studies have reported that propolis administration does not significantly affect milk yield or feed utilization, indicating that variations observed in the present study may be related to changes in udder health status rather than a direct effect of propolis itself (25). Increases in milk fat and lactose content observed in cows treated with the 10% formulation are consistent with earlier reports suggesting that propolis, particularly when used in combination with other bioactive compounds, may be associated with alterations in milk composition (9). However, these changes remained within physiological ranges and were not statistically robust; therefore, they should be interpreted cautiously. The divergent trends in milk yield between the 5 and 10% groups may reflect concentration-related differences in tolerability or formulation characteristics rather than a direct dose–response relationship.

The biochemical parameters evaluated in this study showed fluctuations over the observation period. While previous work by Šuran et al. (1) reported decreases in serum ALT and GGT following topical propolis application in cows with subclinical mastitis, similar trends were not consistently observed in the present study. The positive correlation between creatinine and urea concentrations aligns with findings by Elek et al. (26), who described comparable associations during periods of physiological stress in dairy cattle. Importantly, all biochemical values remained within established reference ranges, suggesting that the applied propolis formulations did not induce detectable hepatic or renal impairment under the conditions of this pilot study.

Changes in leukocyte profiles, including increased lymphocyte counts and a concomitant rise in eosinophil levels—particularly in the 10% treatment group—are consistent with previous observations reported by Cristina et al. (27) and Santos et al. (28) following propolis administration. The present study was not designed to investigate immune mechanisms, and these findings should therefore be interpreted with caution.

An interesting observation was that the 5% propolis emulsion was associated with a greater reduction in bacterial counts compared to the 10% formulation. This finding is consistent with reports by Bankova et al. (29), who emphasized that the biological activity of propolis is not necessarily proportional to its concentration. Factors such as solubility, phytochemical composition, and bioavailability may play a role in modulating biological effects. Nevertheless, due to the limited sample size and lack of randomized controls, this observation should be considered hypothesis-generating and requires confirmation in larger, controlled studies.

An additional novel aspect of this study is the use of alginate as a delivery-enhancing matrix for intramammary propolis administration. To the authors’ knowledge, this is the first *in vivo* study in dairy cows demonstrating that an alginate-based propolis formulation can be applied intramammarily, addressing a gap in previous research where propolis has primarily been evaluated as ethanolic extracts or in non-optimized formulations. Previous studies have primarily evaluated propolis as ethanolic extracts or non-optimized formulations in mastitis-related models, while alginate has been widely applied as a biocompatible delivery matrix in pharmaceutical and biomedical applications (30).

Natural extracts such as propolis represent complex multicomponent systems, and their antioxidant activity depends on multiple reaction mechanisms (31, 32). Consequently, the use of more than one analytical method is recommended for antioxidant assessment (33). In the present study, five spectrophotometric assays were applied to characterize the antioxidant potential of the propolis formulation. Radical scavenging activity followed the order TFPH < DPPH < ABTS, consistent with previous reports (34, 35). Differences between assays can be attributed to methodological factors such as solvent polarity and pH conditions, which influence phenolic compound reactivity (21, 36, 37).

The scientific literature indicates that antioxidant values vary depending on methodology (51, 70). In our findings, the radical scavenging activity of propolis extracts followed the order TFPH < DPPH < ABTS. Similar patterns have been reported (34, 35). The lower value in the TFPH assay may be explained by the use of a strongly acidic medium, which reduces the effectiveness of phenolic compounds (21, 36, 58). The DPPH assay showed limited activity, likely due to its radical solubility only in organic solvents (56, 66), which restricts the measurement of hydrophilic antioxidant activity (45, 64). In contrast, the ABTS’ radical cation is soluble in both aqueous and organic media (45, 67), allowing for evaluation of both hydrophilic and lipophilic antioxidant compounds (37, 51, 52). Reducing power, assessed by CUPRAC and FRAP assays, showed higher values for CUPRAC, likely due to its operation at near-neutral pH, which favors phenolic hydroxyl group dissociation (22, 38, 53, 54). The identification of flavan-3-ols such as (+)-catechin and (−)-epicatechin further supports the antioxidant potential of the tested formulation (39, 40). However, these *in vitro* findings serve to characterize the chemical properties of the formulation and should not be extrapolated to *in vivo* antioxidant or clinical effects.

The phenolic profile analysis confirmed the presence of multiple bioactive compounds in the 5% propolis alginate solution. Phenolic acids and flavonoids are widely recognized as major contributors to the biological properties of propolis (41, 42). While the antioxidant and anti-inflammatory properties of phenolic compounds have been documented in various experimental contexts (43, 44), the present study does not allow conclusions regarding their specific mechanisms of action in mastitis.

The antioxidant activity of phenolic compounds is closely related to anti-inflammatory (43, 44, 57, 62), antidiabetic (63, 65), cardiovascular (59, 48), and other important biological effects (60, 61). Enhancing the solubility and delivery of natural compounds is considered an important step in translating their biological properties into practical applications (30). The development of propolis–alginate formulations represents a promising approach to improve local delivery and retention of active compounds. Nevertheless, the present findings should be interpreted as preliminary. Further research employing adequately powered, randomized, and controlled study designs with extended follow-up periods is required to validate efficacy, assess safety, and compare propolis-based formulations with standard intramammary therapies.

## 5 Conclusion

A 5% propolis concentration shows greater efficacy than 10%, likely due to improved absorption. These findings highlight propolis as a promising natural alternative, though further research is needed to refine dosing and assess long-term safety.

## Data Availability

The raw data supporting the conclusions of this article will be made available by the authors, without undue reservation.
